# Macrohormones in endocrinology: prevalence, clinical impact, and diagnostic approaches

**DOI:** 10.3389/fendo.2026.1913010

**Published:** 2026-07-27

**Authors:** Agata Bronisławska, Olivia Cyran-Stańczak, Robert Bronisławski, Alicja Solecka, Agata Berlińska, Renata Świątkowska-Stodulska, Jarosław Piotr Jendrzejewski

**Affiliations:** 1 Student Scientific Association of Endocrinology, Faculty of Medicine, Medical University of Gdansk, Gdańsk, Poland; 2 Department of Endocrinology and Internal Medicine, Faculty of Medicine, Medical University of Gdańsk, Gdańsk, Poland; 3 Department of Endocrinology and Internal Medicine, University Clinical Center, Gdańsk, Poland

**Keywords:** macrocomplexes, macrohormone assay, macromolecules, macroprolactin, macro-TSH

## Abstract

Macrohormones also called macromolecules or macrocomplexes are described as an antigen-antibody complex, consisting of monomeric hormones bound to immunoglobulins. These molecules exhibit minimal or no biological activity. However, their presence may falsely indicate elevation of active hormones levels which can lead to significant diagnostic challenges. Currently, polyethylene glycol precipitation (PEG) is one of the most recognized methods for identifying macrohormones and it has been widely used in diagnosing macroprolactinemia as awareness of its clinical implications is well recognized. Macrohormone assessment is particularly recommended when clinical symptoms are incompatible with laboratory findings, as it may help avoid unnecessary diagnostic procedures and treatments. Recently, there has been an increasing number of reports highlighting diagnostic challenges associated with other macrohormones, including macro-TSH, macro-FSH, macro-LH, macro-PTH, macro-GH, macro-ACTH, macro-insulin, and macro-calcitonin. The presence of these molecules can lead to overdiagnosis. This review aims to summarize the latest findings and highlight the importance of considering the presence of macrohormones in patients with discrepancies between clinical presentation and hormonal laboratory tests.

## 1 Introduction

### 1.1 Background

Macrohormones also referred to as hormone macrocomplexes or high-molecular-weight hormone molecules constitute an important and underrecognized source of diagnostic error in endocrine practice. These circulating entities most commonly represent immune complexes formed by monomeric hormones bound to immunoglobulins, resulting in high-molecular-weight particles that typically exhibit minimal or no biological activity. Nevertheless, macrohormones retain immunoreactivity and are detected by routine immunoassays, producing spuriously false elevated hormone concentrations results that may be mistaken for true endocrine disease. In an era of widespread automated hormone testing, this discordance between laboratory results and clinical phenotype has become increasingly relevant, with potential consequences ranging from prolonged diagnostic work-up to inappropriate therapy and avoidable harm.

The concept of macrohormones was first brought to broader clinical attention through macroprolactinemia, described in 1985 as persistent prolactin elevation in the absence of typical clinical manifestations (1). Since then, nomenclature has remained inconsistent, reflecting ongoing discovery and incomplete biochemical characterization of these complexes. Prefixes such as “macro-,” “big-,” and “big-big-” have been used to denote larger circulating forms, and terms such as “high-molecular-weight” or “large-molecular-weight” hormones appear interchangeably across the literature (2, 3). This lack of standardized terminology complicates systematic synthesis of macrohormones prevalence and may contribute to underestimation in review reports, hence in daily clinical practice. When referring to macrohormones, we suggest using the hyphenated form (e.g., macro-thyrotropin, macro-TSH, macro-insulin), as this is the form most commonly used in the existing literature. The exception is macroprolactin, a term established over 40 years ago and applied consistently in this non-hyphenated form since.

Despite heterogeneity among individual hormones, macrohormones share several common features. Their large molecular size is thought to limit transcapillary passage and reduce access to target tissues, while antibody binding may sterically hinder receptor interaction, collectively resulting in markedly reduced *in vivo* bioactivity (4–9). At the same time, immunoassay detection of macrohormones alongside monomeric hormones may generate clinically misleading results. Accordingly, macrohormones should be considered whenever hormone concentrations are incongruent with the patient’s symptoms, or associated biochemical markers. Failure to recognize immunoassay interference may lead to unnecessary imaging studies, repeated laboratory testing, dynamic endocrine testing, and inappropriate pharmacological or surgical interventions.

The epidemiology of macrohormones remains incompletely defined. Macroprolactinemia is the best-studied entity and is reported in 10-46% patients with elevated prolactin concentrations (10). In contrast, most other macrohormones have been described primarily through case reports and small series, limiting robust estimates of prevalence and clinical impact. Nonetheless, an expanding body of literature highlights that macrocomplex formation is not confined to prolactin and may involve a broad range of endocrine analytes, including thyrotropin, gonadotropins, parathyroid hormone, growth hormone, adrenocorticotropic hormone, insulin, and calcitonin (5, 11–16).

In this review, we synthesize current evidence on macrohormones across endocrinology, focusing on (i) their biochemical characteristics and proposed mechanisms of formation, (ii) the diagnostic pitfalls and methodological limitations of commonly used laboratory approaches, and (iii) the implications for clinical decision-making and patient safety. By integrating endocrine and laboratory perspectives, we aim to provide a practical framework for recognizing macrohormone-related immunoassay interference and to highlight priorities for future research, including standardization of terminology and diagnostic algorithms.

### 1.2 General overview of macrohormones

Macrohormones are high molecular weight complexes of a monomeric hormone and an immunoglobulin, which accumulate in serum due to their delayed clearance. These complexes are biologically inactive, but still detectable by routine immunoassays, so their presence causes spuriously elevated results inconsistent with the clinical picture - representing a form of immunoassay interference rather than real disease. Macrohormones must be distinguished from other high molecular weight hormone variants, such as hormone precursors and other “big” hormone forms, since only the biologically inactive, antibody-bound complex meets the precise definition of a macrohormone.

In most reported cases involving these molecules, immunoglobulin G is the dominant binding partner, although complexes involving IgA and, rarely, other isotypes have also been described (7, 17). In addition to classical antigen-antibody complexes, macrohormones may also originate from hormone self-association driven by post-translational modifications that promote aggregation, such as glycosylation and phosphorylation (6). This biochemical diversity likely contributes to the heterogeneity observed across macrohormones in terms of immunoreactivity, assay susceptibility, and persistence over time.

A defining clinical feature of macrohormones is the frequent dissociation between markedly abnormal laboratory measurements and minimal or absent biological effects. Several mechanisms may account for reduced *in vivo* activity. First, the increased molecular size of macrocomplexes is thought to limit transcapillary passage, thereby reducing hormone access to target tissues; this mechanism has been most extensively discussed in macroprolactinemia (9). Second, antibody binding may sterically hinder receptor interaction or alter hormone conformation, limiting biological signaling (4). Importantly, despite their reduced bioactivity, macrohormones typically retain immunoreactivity and are therefore detected by routine immunoassays alongside monomeric hormone, potentially producing spurious elevations that mimic true endocrine disease (5, 8). Consequently, macrohormone-related interference should be considered whenever hormonal results are incongruent with the clinical phenotype, associated biochemical markers, or imaging findings.

### 1.3 Analytical approaches to macrohormone detection

**Figure 1:**
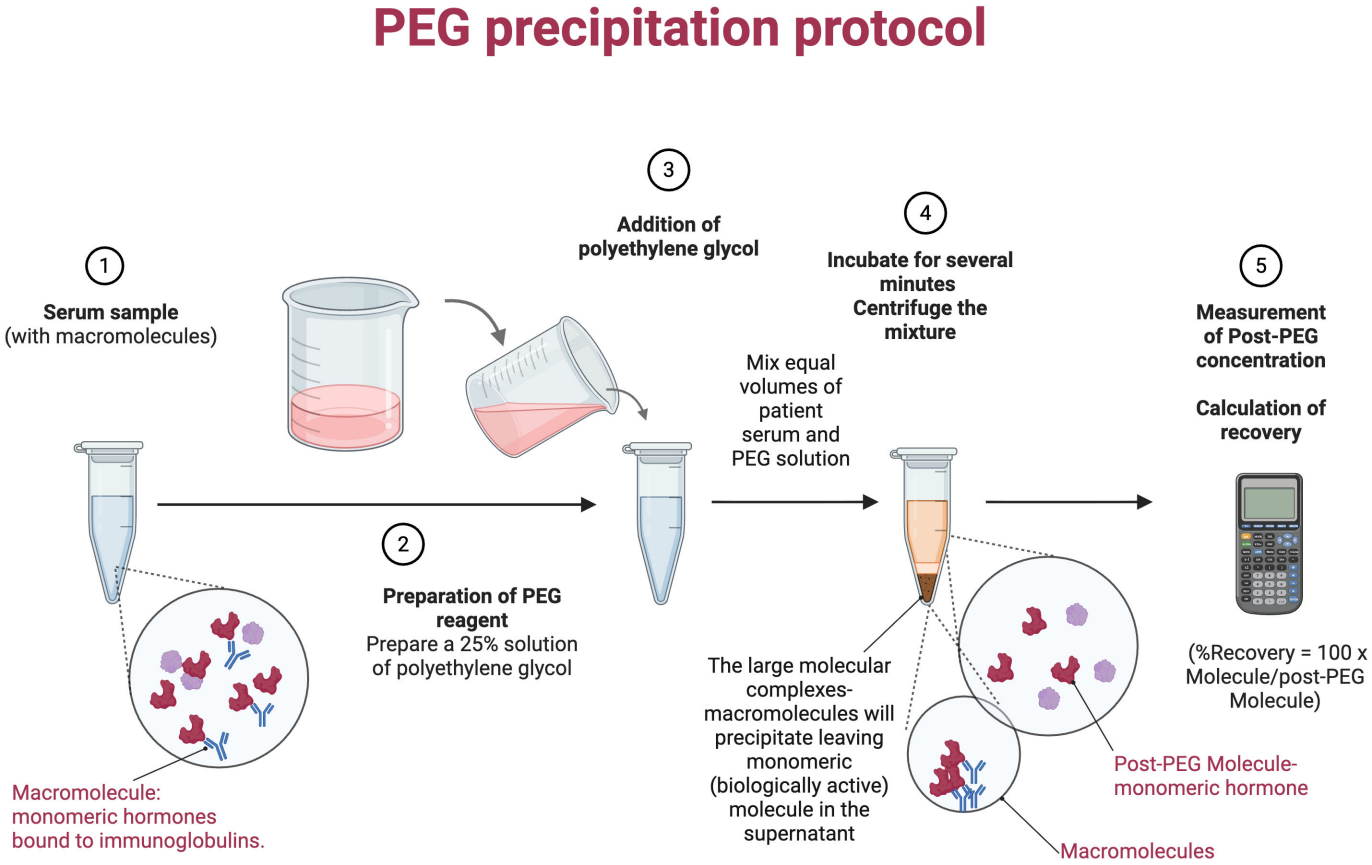
Polyethylene glycol precipitation protocol. Created in BioRender. Berlińska, A. (2026). https://BioRender.com/0nrzdst.

In routine practice, evaluation of suspected macrohormones follows a stepwise approach that distinguishes screening tests from confirmatory methods. Polyethylene glycol (PEG) precipitation remains the most widely available screening technique and is used routinely in the evaluation of macroprolactin. PEG promotes precipitation of high-molecular-weight proteins based on solubility changes, thereby enriching the supernatant for monomeric hormone (18). Following precipitation, hormone concentration is reassessed, and the result is interpreted as a surrogate marker of high-molecular-weight interference (**Figure 1**).

However, PEG precipitation has important limitations. Partial precipitation of monomeric hormone may occur, leading to underestimation of the biologically active fraction; for prolactin, post-PEG values may be underestimated by up to ~25% (19). In addition, residual PEG in the sample matrix may affect certain immunoassay platforms, contributing to method-dependent variability (10). Therefore, interpretation should not rely solely on percent recovery but should incorporate the absolute post-PEG hormone concentration in relation to assay-specific reference intervals (20). This is particularly relevant because macrohormone interference may coexist with genuine elevation of monomeric hormone, producing intermediate recoveries that remain clinically meaningful.

In clinical practice, PEG recovery is calculated as the post-precipitation hormone concentration expressed as a percentage of the pre-PEG value. Across the macrohormone literature, no universal recovery threshold has been established. A cutoff of <40% is frequently applied for macroprolactin screening, although alternative thresholds (<60% or <30%) have been used (10). Importantly, recovery thresholds cannot be extrapolated across hormones, because PEG-induced co-precipitation of monomeric analyte varies substantially between assays and hormone classes. For example, TSH may be more prone to PEG precipitation than prolactin, necessitating different interpretive cutoffs in macro-TSH evaluation (21).

Since PEG precipitation protocols differ considerably across laboratories, various amounts of both the macrohormone complex and the genuine monomeric hormone can be expected to be precipitated (6, 22). In addition, the commercial analytical platforms used across laboratories may yield different results for the same post-PEG samples, meaning that a universal cutoff does not exist, and platform-specific validation is required (22). Laboratories must therefore establish their own method- and assay-specific post-PEG reference intervals. Collectively, these considerations highlight that PEG precipitation is best viewed as a pragmatic screening tool that requires assay- and analyte-aware interpretation rather than a definitive diagnostic test.

Gel filtration chromatography (GFC) (also referred to as size-exclusion chromatography) remains the reference method for confirming macrohormones (18, 23). By separating circulating species by molecular size, GFC enables direct demonstration of a high-molecular-weight immunoreactive peak distinct from the monomeric hormone fraction and allows semiquantitative estimation of macrohormone contribution. Nevertheless, GFC is time-consuming, labor-intensive, and costly, and requires specialized equipment and expertise, limiting availability in routine clinical laboratories.

Several alternative or adjunctive approaches have been reported. Pretreatment with protein A or protein G with subsequent ultrafiltration may reduce apparent hormone concentrations by removing IgG-bound complexes and can support an immunoglobulin-mediated mechanism. Similarly, anti-human IgG immunoprecipitation can provide evidence for IgG involvement. These methods, however, may be less informative in IgA-associated complexes or in non-immune-complex high-molecular-weight molecules. Ultrafiltration has also been explored but, across reported studies, has shown weaker correlation with GFC than PEG precipitation (19). Finally, heterophilic antibody-blocking reagents may be helpful when differential diagnosis includes heterophilic antibody interference, which can also cause spuriously elevated results in immunoassays. These reagents do not detect macrocomplexes per se but can help exclude a major alternative source of analytical artifact (13) (**Table 1**).

**Table 1:** Summary of macromolecules described in literature.

Macromolecule	Composition	Prevalence/cases	Clinical impact	Detection methods	References
Macroprolactin (macro-PRL)	Prolactin + IgG (sometimes IgA or other molecules)	10–46% of hyperprolactinemia; ~0.2–4% in general population	Falsely elevated PRL; usually asymptomatic; risk of misdiagnosis as prolactinoma	PEG precipitation (<40–60% recovery), GFC gold standard	(1, 10, 20, 34–37)
Macro-thyrotropin (macro-TSH)	TSH + IgG (rarely IgA)	0.6–1.6% of patients with TSH >10 mIU/L; rare in neonates (maternal transfer)	Mimics subclinical hypothyroidism; unnecessary levothyroxine therapy risk	PEG precipitation (<25% recovery), GFC or radiolabeled TSH binding assay	(21, 47–51, 55–58)
Macro-follitropin (macro-FSH)	FSH + IgG	~ 6 cases	Falsely elevated FSH; can mimic ovarian failure/POI, misdiagnosis of infertility	PEG precipitation (<20–30% recovery), GFC	(5, 66, 70, 71)
Macro-luteinizing hormone (macro-LH)	LH + IgG	~ 5 cases	Falsely elevated LH; can mimic PCOS or pituitary disorder	PEG precipitation (<10% recovery), GFC, immunoprecipitation with protein G	(14, 65, 69, 72)
Macro-parathyroid hormone (macro-PTH)	PTH + IgG	~ 5 cases	Falsely elevated PTH; may mimic primary/secondary hyperparathyroidism	PEG precipitation (<10% recovery), GFC	(12, 76, 77, 79)
Macro-growth hormone (macro-GH)	GH + IgG	Only 2 cases (2023, 2026)	Falsely elevated GH; may mimic acromegaly despite normal IGF-1	PEG precipitation (<20% recovery), GFC	(13, 86)
Macro-adrenocorticotropin (macro-ACTH)	ACTH + IgG	Only 2 cases (2023)	Falsely elevated ACTH; can mimic Cushing’s or adrenal insufficiency	PEG precipitation, GFC	(15, 83, 87–89)
Macro-insulin	Insulin + IgG	Only 1 case (2024)	Falsely elevated insulin; mimics hyperinsulinemia without hypoglycemia	PEG precipitation, assay comparison	(16)
Macro-calcitonin	Calcitonin + immunoglobulin	Only 3 cases (2015)	Falsely elevated calcitonin; mimics persistent or recurrent MTC	PEG precipitation, GFC	(11)

**Figure 2:**
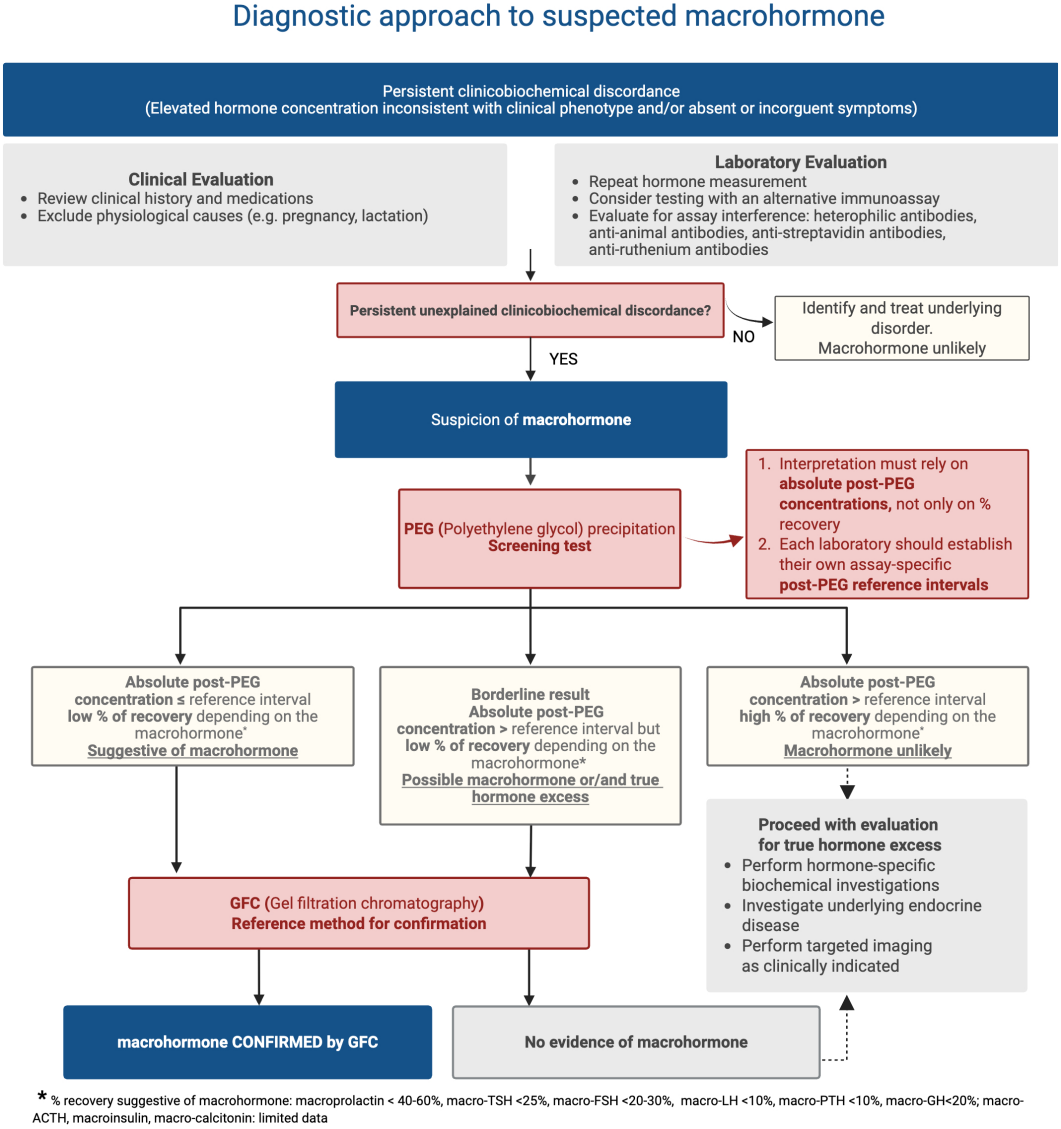
Diagnostic approach to suspected macrohormone. Created in BioRender. Berlińska, A. (2026). https://BioRender.com/od4ycpc.

Overall, a practical diagnostic strategy integrates clinical assessment with sequential laboratory evaluation: (i) confirmation of discordance and exclusion of common physiological/pathological explanations, (ii) assessment of analytical plausibility through repeat testing and/or cross-platform comparison when feasible, (iii) targeted screening for high-molecular-weight interference (most commonly PEG precipitation), and (iv) confirmatory chromatographic characterization when results are consequential for management and resources permit (**Figure 2**).

### 1.4 Analytical interferences

From an analytical perspective, macrohormones are considered a form of immunoassay interference. However, unlike other antibody-associated interferences, such as heterophilic antibodies, anti-animal antibodies, anti-streptavidin antibodies, or anti-ruthenium antibodies, macrohormones are genuine *in vivo* hormone–autoantibody complexes rather than artifacts arising from interactions between endogenous antibodies and assay reagents during the analytical procedure. These antibody-mediated interferences can be excluded by using blocking reagents, testing on an alternative assay platform or performing serial dilutions (24, 25).

Although macrohormones typically lead to falsely elevated hormone concentrations, clinicians should also be aware of analytical artifacts that produce falsely low results. The most important example is the hook effect (also known as the prozone phenomenon), which may occur in two-site (sandwich) immunoassays (26). This phenomenon has also been described in prolactin measurement (27). It occurs when an extremely high analyte concentration saturates both the capture and detection antibodies independently, preventing formation of the antibody–analyte–antibody sandwich required for signal generation (26). Consequently, the assay signal is disproportionately reduced, resulting in a falsely low measured concentration. When the hook effect is suspected, the sample should be serially diluted and the measurement repeated (26). Although confirmation of the hook effect is technically straightforward, its recognition depends on close communication between the clinician and the laboratory.

### 1.5 Methods (literature search strategy)

This review is based on a comprehensive literature search focused on macrohormones in endocrinology. Relevant publications were identified through searches of major bibliographic databases, including PubMed, Scopus, and Google Scholar. Search terms included combinations of hormone names with descriptors such as “macro,” “big,” “big-big,” “high-molecular-weight,” and “large-molecular-weight.” No restrictions were applied with respect to publication date or study design to maximize capture of case reports, series, and methodological studies.

## 2 Macrohormones

### 2.1 Macroprolactin

Macroprolactin (macro-PRL) is the most extensively characterized macrohormone and represents the prototypical example of clinically relevant immunoassay interference in endocrinology. Among patients evaluated for hyperprolactinemia, macroprolactinemia accounts for approximately 10-46% of cases, whereas its prevalence in the general population is estimated at 0.2-4% in women and up to 4.5% in men (20).

Prolactin (PRL) is a peptide hormone secreted by lactotroph cells of the anterior pituitary and plays a central role in mammary gland development, lactation, and modulation of the hypothalamic-pituitary-gonadal axis by suppression of gonadotropin secretion (28, 29). Measurement of serum prolactin is routinely performed in the evaluation of menstrual disturbances, infertility, galactorrhea, hypogonadism, and pituitary disorders. Elevated prolactin concentrations may reflect physiological states, pharmacological effects, pituitary pathology, or analytical interference, most notably due to the presence of macro-PRL (30).

Circulating PRL exists in several molecular forms. Monomeric prolactin (23 kDa) constitutes approximately 80-95% of total serum prolactin and represents the biologically active fraction. Dimeric prolactin (“big PRL,” 48–56 kDa) accounts for 5-10%, whereas macroprolactin (“big-big PRL”), typically exceeding 150 kDa, consists of prolactin bound predominantly to immunoglobulin G, although IgA-associated complexes have also been described (31). So called “macroprolactinemia” is commonly defined by a predominance of macro-PRL, usually exceeding 60% of total circulating prolactin (10).

The biological activity of macro-PRL is markedly reduced or absent. Proposed mechanisms include impaired transcapillary passage due to molecular size and reduced receptor-binding capacity (4, 9). Despite its limited bioactivity, macro-PRL is readily detected by standard immunoassays, resulting in falsely elevated total prolactin concentrations and potential diagnostic confusion. Consequently, macro-PRL should be suspected when prolactin levels are elevated in the absence of typical clinical manifestations of hyperprolactinemia.

Although macroprolactinemia is often considered clinically benign, its coexistence with monomeric hyperprolactinemia has been documented and may obscure interpretation of laboratory results (32). In *in vitro* models, dissociation of prolactin-immunoglobulin complexes has been reported (33). These observations underscore the importance of deep biochemical characterization together with the clinical presentation.

The first well-documented case of macroprolactinemia was described in 1985 by Jackson et al. in a 35-year-old nonpregnant woman with persistently increased serum prolactin concentrations (350–400 ng/mL) over a three-year period (1). Despite marked hyperprolactinemia, computed axial tomography showed no evidence of a pituitary adenoma. Endocrine profiling demonstrated attenuation of the normal nocturnal rise in prolactin during 24-hour sampling, preserved suppression of prolactin secretion with low-dose dopamine infusion, and a blunted prolactin response to a thyrotropin-releasing hormone bolus. Subsequent gel filtration chromatography identified macroprolactin as the predominant circulating immunoreactive form, accounting for the discordantly high measured prolactin concentrations.

Since its initial description by Jackson et al. in 1985, macroprolactinemia has been repeatedly shown to result in unnecessary diagnostic procedures and inappropriate treatment, including pituitary imaging and dopamine agonist therapy (34–36). Polyethylene glycol precipitation is widely used as a screening method, whereas gel filtration chromatography remains the reference technique for definitive confirmation (19, 37). Current Endocrine Society guidelines recommend testing for macro-PRL in patients with asymptomatic hyperprolactinemia (38).

Emerging data suggest that macro-PRL may not be entirely biologically inert. Pilot studies have reported associations with reproductive dysfunction, cardiometabolic risk factors, mood disturbances, platelet activation, and autoimmune disease activity such as systemic lupus erythematosus, although causality remains unproven and further investigation is required (39–44). Transplacental transfer of prolactin-IgG complexes has also been demonstrated, resulting in transient macroprolactinemia in neonates born to affected mothers (45).

Macro-PRL is a frequent and clinically important cause of hyperprolactinemia. Its recognition is essential to avoid misdiagnosis, unnecessary investigations, and inappropriate therapy. Although routine screening practices vary, increasing awareness of macro-PRL has substantially improved diagnostic accuracy and patient management. Continued research is needed to clarify its long-term clinical significance and to refine standardized diagnostic algorithms.

### 2.2 Macro-thyroid-stimulating hormone

Macro-thyroid-stimulating hormone (macro-TSH) is an uncommon but clinically relevant cause of persistently elevated serum TSH concentrations in euthyroid individuals and represents an important source of immunoassay interference in thyroid function testing. Given the widespread use of TSH measurement as the primary screening tool for thyroid dysfunction, failure to recognize macro-TSH may result in misdiagnosis and inappropriate initiation of levothyroxine therapy.

Thyrotropin is a glycoprotein hormone secreted by the anterior pituitary in response to thyrotropin-releasing hormone and plays a central role in regulating thyroid hormone synthesis and secretion. In clinical practice, elevated TSH concentrations with normal circulating free thyroxine define subclinical hypothyroidism, a condition affecting up to 10% of adults (46, 47). In general, American Thyroid Association and European Thyroid Association guidelines recommend levothyroxine therapy in asymptomatic individuals with TSH levels exceeding 10 mIU/L, particularly in younger patients (48, 49). In this context, analytical interference must be excluded, as unnecessary treatment may lead to iatrogenic thyrotoxicosis (50, 51).

Macro-TSH consists of monomeric TSH complexed with immunoglobulins, most commonly IgG, although IgA-associated complexes have also been reported (52). These high-molecular-weight complexes, typically exceeding 150 kDa (monomeric TSH has molecular mass of 28 kDa), exhibit markedly reduced biological activity but are detected by routine immunoassays, leading to spuriously elevated TSH concentrations (53–55). Due to their prolonged circulatory half-life and impaired clearance, macro-TSH complexes accumulate in serum, while free thyroid hormone levels remain within reference ranges.

The reported prevalence of macro-TSH among patients with markedly elevated TSH concentrations (>10 mIU/L) ranges from approximately 0.6% to 1.6% (53, 56). Although rare, macro-TSH closely mimics subclinical hypothyroidism and has been repeatedly associated with unnecessary diagnostic procedures and inappropriate levothyroxine therapy. Longitudinal observations suggest that macro-TSH may persist for years, although spontaneous resolution has been documented in a minority of cases (57).

Macro-TSH was first described in 1981 by Spitz et al, when a euthyroid individual with persistently elevated TSH was found to harbor a high-molecular-weight TSH complex with minimal biological activity despite preserved receptor binding (58). Since then, numerous case reports have highlighted the diagnostic challenges posed by macro-TSH, particularly in asymptomatic patients with unexplained TSH elevation and normal thyroid hormone concentrations (50, 51, 55).

A distinct clinical scenario in which macro-TSH may have important implications is neonatal screening for congenital hypothyroidism. Transplacental transfer of maternal anti-TSH autoantibodies can result in the formation of macro-TSH in neonates, leading to markedly elevated TSH levels on dried blood spot screening despite normal thyroid function. In such cases, TSH concentrations typically normalize over time as maternal immunoglobulins are cleared, underscoring the importance of recognizing macro-TSH to avoid unnecessary treatment during infancy (59–61).

Polyethylene glycol precipitation is the most used screening method for macro-TSH in clinical practice. However, interpretation of PEG recovery requires caution, as monomeric TSH is more susceptible to PEG-induced precipitation than prolactin. Consequently, higher recovery thresholds have been proposed for macro-TSH detection. Recent systematic analyses suggest that PEG-precipitable TSH fractions exceeding 75% are more indicative of macro-TSH, whereas lower cutoffs may lead to false-positive results (21, 62, 63). Gel filtration chromatography remains an important confirmatory method, although weak antibody-hormone binding may result in partial dissociation of macro-TSH complexes during analysis, potentially yielding false-negative findings (60, 64).

In summary, in patients with persistent TSH elevation, normal free thyroid hormones, and an absence of clinical features of hypothyroidism, evaluation for macro-TSH should be considered as part of a systematic approach to immunoassay interference.

### 2.3 Macro-gonadotropins: macro-FSH and macro-LH

Macro-follicle-stimulating hormone (macro-FSH) and macro-luteinizing hormone (macro-LH) represent rare but clinically relevant forms of immunoassay interference involving pituitary gonadotropins. Both entities consist of high-molecular-weight complexes formed by binding of the native hormone to circulating immunoglobulins, most commonly immunoglobulin G, resulting in spuriously elevated serum gonadotropin concentrations with minimal or absent biological activity (65, 66).

Follicle-stimulating hormone and luteinizing hormone are glycoproteins secreted by gonadotroph cells of the anterior pituitary and play central roles in reproductive physiology, including folliculogenesis, ovulation, spermatogenesis, and steroidogenesis (67, 68). Measurement of FSH and LH is routinely used in the evaluation of infertility, menstrual irregularities, pubertal disorders, and gonadal failure. Physiological elevations of FSH and LH occur during the menopausal transition and in primary gonadal insufficiency. In addition, altered gonadotropin profiles are characteristic of polycystic ovary syndrome, typically reflecting an increased LH/FSH ratio rather than absolute gonadotropin excess. In these settings, discordantly elevated gonadotropin concentrations should prompt consideration of analytical interference.

Macro-FSH and macro-LH are composed of monomeric gonadotropins complexed with immunoglobulins, forming circulating macromolecular species exceeding 150 kDa. These complexes are cleared slowly from the circulation and are readily detected by standard immunoassays, resulting in falsely elevated hormone concentrations. Despite preserved immunoreactivity, their biological activity is markedly reduced, explaining the absence of clinical features consistent with gonadal failure or gonadotropin excess in affected individuals (5, 69).

To date, only a limited number of cases of macro-FSH and macro-LH have been reported. Across these reports, a consistent pattern emerges: markedly elevated FSH or LH concentrations that are incompatible with the patient’s reproductive history, hormonal milieu, or imaging findings. In women, macro-FSH has been described in the context of preserved ovarian function, normal estradiol and anti-Müllerian hormone concentrations, regular menstrual cycles, or spontaneous conception, thereby mimicking primary ovarian insufficiency (5, 66, 70, 71). Similarly, macro-LH has been reported in patients with menstrual irregularities or features suggestive of polycystic ovary syndrome, but with gonadotropin concentrations far exceeding those typically observed in these conditions (65, 69, 72).

Polyethylene glycol precipitation has been used as a screening tool for both macro-FSH and macro-LH, demonstrating low post-precipitation recovery <30% and <10% for macro-FSH and macro-LH, respectively (**Table 1**). Gel filtration chromatography, high-performance liquid chromatography, and immunoglobulin-binding assays have been used in selected cases to confirm the macromolecular nature of these complexes and to identify IgG as the predominant binding partner (65, 71). Notably, assay interference due to macro-gonadotropins has led to unnecessary investigations, including pituitary imaging, genetic testing for gonadal failure, and inappropriate counseling regarding infertility or premature menopause. Interestingly, Nyshiyama et al. reported a case of coexisting macro-TSH and macro-LH in a 60-year-old male. Cross-reactivity analyses showed that the patient’s autoantibodies exhibited the highest reactivity with TSH and LH and partial reactivity with FSH, hCG, and hCGβ, likely due to structurally homologous cystine-knot domains shared across glycoprotein hormones (14).

The biological significance of macro-FSH and macro-LH remains incompletely understood. While these macromolecules are generally considered biologically inactive, isolated reports have suggested that the potential associations with reproductive dysfunction caused by presence of anti-FSH antibodies (5, 66, 73). However, a causal relationship has not been established, and current evidence supports the interpretation of macro-gonadotropins primarily as a source of analytical interference rather than true endocrine pathology.

Macro-FSH and macro-LH are rare causes of falsely elevated gonadotropin concentrations that may closely mimic disorders such as primary ovarian insufficiency or polycystic ovary syndrome. Awareness of macro-gonadotropins can prevent misdiagnosis, particularly in the evaluation of infertility and menstrual disorders.

### 2.4 Macro-parathyroid hormone

Macro-parathyroid hormone (macro-PTH) is a recently recognized and rare cause of falsely elevated serum parathyroid hormone concentrations and represents an emerging form of immunoassay interference in disorders of calcium-phosphate metabolism. Although markedly elevated PTH levels typically suggest primary or secondary hyperparathyroidism, the identification of macro-PTH is critical in patients in whom biochemical findings are discordant with the clinical picture. Pseudohypoparathyroidism should also be considered in the differential diagnosis of elevated PTH concentrations; however, unlike macro-PTH, it is characterized by biochemical evidence of PTH resistance, including hypocalcemia and hyperphosphatemia (74).

Parathyroid hormone is a peptide hormone secreted by the parathyroid glands and plays a central role in calcium and phosphate homeostasis through its actions on bone, kidney, and indirectly the intestine (75). In clinical practice, elevated PTH concentrations are most encountered in the setting of hypercalcemia in primary hyperparathyroidism or hypocalcemia in secondary hyperparathyroidism. In contrast, disproportionately elevated PTH concentrations in the presence of normocalcemia (after albumin adjustment), normal phosphate and vitamin D levels, preserved renal function, and unremarkable parathyroid imaging should prompt consideration of analytical interference, including macro-PTH.

Macro-PTH represents a high-molecular-weight complex formed by binding of monomeric PTH to circulating immunoglobulins, most commonly immunoglobulin G. These complexes are biologically inactive or minimally active but are readily detected by routine immunoassays, leading to spuriously elevated total PTH concentrations. Due to impaired renal clearance, macro-PTH accumulates in the circulation, whereas calcium-phosphate homeostasis remains unaffected (76).

The term macro-PTH was first introduced in a conference report by Prodan et al. in 2016 describing an asymptomatic patient with extreme PTH elevation despite normal biochemical and imaging findings (77). Since then, a small number of case reports have confirmed macro-PTH as a distinct cause of isolated PTH elevation (12, 76, 78, 79). Review of reported cases indicates a largely consistent clinical profile, characterized by markedly elevated PTH concentrations, normocalcemia, absence of skeletal or renal complications of hyperparathyroidism, and lack of structural parathyroid disease on imaging. In several cases, macro-PTH was initially misinterpreted as normocalcemic primary hyperparathyroidism or vitamin D-related secondary hyperparathyroidism, resulting in prolonged follow-up or unnecessary diagnostic evaluation (12, 76).

Polyethylene glycol precipitation has proven to be a useful screening tool for macro-PTH, typically demonstrating very low post-precipitation PTH recovery, in all reported cases less than 35%. Size-exclusion chromatography remains the reference method for definitive confirmation; however, its limited availability has restricted its use in routine clinical practice. Importantly, serial dilution testing and heterophilic antibody-blocking reagents are usually unremarkable in macro-PTH-associated interference, underscoring the need for targeted investigation when macro-PTH is suspected (76).

Macro-PTH should be clearly distinguished from parathyroid hormone-related peptide (PTHrP), which is a structurally distinct molecule produced independently of the parathyroid glands. Although PTHrP shares partial sequence homology with PTH, it does not form immunoglobulin complexes and retains full biological activity. PTHrP is associated with malignancy-related hypercalcemia and increased bone resorption, in contrast to the clinically silent biochemical profile observed in macro-PTH-associated assay interference (80–82).

In conclusion, macro-PTH is an uncommon but clinically relevant cause of isolated PTH elevation that may closely mimic hyperparathyroid states in laboratory testing. Given the limited number of reported cases, further studies are required to better define the prevalence, molecular characteristics, and clinical implications of macro-PTH in endocrine practice.

### 2.5 Rare pituitary macrohormones: macro-growth hormone and macro-adrenocorticotropic hormone

Macro-growth hormone (macro-GH) and macro-adrenocorticotropic hormone (macro-ACTH) are exceptionally rare causes of immunoassay interference involving pituitary hormones. Both entities are characterized by the formation of high-molecular-weight complexes between the native hormone and circulating immunoglobulins, resulting in spuriously elevated hormone concentrations with little or no biological activity. Although infrequently encountered, failure to recognize these macrohormones may lead to significant diagnostic and therapeutic consequences (13, 15, 83).

Growth hormone and adrenocorticotropic hormone are peptide hormones secreted by the anterior pituitary and play essential roles in regulation of growth, metabolism, and stress response (84, 85). Measurement of GH after glucose delivery is fundamental in the evaluation of acromegaly, whereas ACTH assessment is essential in the diagnostic work-up of Cushing syndrome and adrenal insufficiency. In both settings, interpretation of isolated hormone elevations must be guided by the clinical context, associated biochemical markers, and imaging findings.

Macro-GH consists of growth hormone bound to immunoglobulins, forming circulating complexes that are detected by certain immunoassays but exhibit minimal biological activity. To date, only two cases of macro-GH have been reported. In 2023 Stelmachowska-Banas et al. described a 61-year-old woman with a pituitary macroadenoma who displayed markedly elevated GH level with normal insulin-like growth factor-1 levels, absence of acromegalic features, and lack of biochemical response to somatostatin analog therapy. PEG precipitation revealed GH recovery rate of 12%. Subsequently, size-exclusion chromatography confirmed a high-molecular-weight of GH complex (670–158 kDa), distinct from monomeric GH (22 kDa) observed in the controls (13). In 2026 Ohno et al. conducted the screening for macro-GH among 185 female patients with elevated GH level. PEG and GFC identified five patients with potential macro-GH, in four patients the cause was due to human anti-rabbit IgG antibody interference. Only one patient (1/185; 0.54%) had macro-GH due to anti-human GH autoantibody (86).

Macro-ACTH represents an immunoglobulin-bound, biologically inactive form of ACTH that can cause isolated ACTH elevation in the absence of cortisol excess or deficiency. Macro-ACTH should not be confused with big-ACTH, as these two entities differ fundamentally in their pathophysiology, clinical significance, and diagnostic approach. Big-ACTH consists of high-molecular-weight ACTH precursors generated by impaired processing of proopiomelanocortin (POMC) in pituitary or ectopic ACTH-secreting tumors (87–89). These precursor peptides retain partial biological activity and therefore indicate an underlying neoplastic process.

In contrast, macro-ACTH is a biologically inactive hormone–immunoglobulin complex that represents a source of immunoassay interference rather than a marker of ACTH-producing tumors. Because big-ACTH is a POMC processing intermediate, its molecular weight is relatively low (approximately 20–30 kDa), comparable to that of monomeric prolactin (23 kDa) and TSH (28 kDa) (90, 91). Consequently, unlike macro-ACTH, big-ACTH would not be expected to precipitate following PEG treatment. GFC therefore remains the most reliable method for distinguishing these two high-molecular-weight forms of ACTH by directly demonstrating their molecular size. Big-ACTH elutes at a molecular weight greater than native ACTH but substantially lower than macro-ACTH, which circulates as an immunoglobulin-bound complex.

This distinction has important practical implications. In the only published case of macro-ACTH, involving a 63-year-old Caucasian man, markedly elevated ACTH concentrations were discordant with the clinical presentation and normal cortisol concentrations. Following PEG precipitation, ACTH recovery was approximately 6%, supporting the presence of macro-ACTH (83).

The clinical impact of macro-GH and macro-ACTH lies primarily in their potential to mimic serious endocrine disorders. Misinterpretation of macro-GH may result in unnecessary pharmacological treatment or surgical intervention for presumed acromegaly, whereas failure to recognize macro-ACTH may lead to extensive diagnostic evaluation for Cushing syndrome or adrenal insufficiency, including invasive procedures.

### 2.6 Macro-insulin

Macro-insulin represents a rare form of immunoassay interference resulting from the formation of high-molecular-weight complexes between endogenous insulin and circulating autoantibodies. Consequently, markedly elevated insulin concentrations may be measured despite the absence of corresponding biological effects, posing a diagnostic challenge in the evaluation of hyperinsulinemia (16).

Macro-insulin should be interpreted in the context of Insulin Autoimmune Syndrome (IAS, Hirata disease), as both entities involve circulating insulin–autoantibody complexes. IAS is a clinical syndrome characterized by spontaneous hyperinsulinemic hypoglycemia, markedly elevated immunoreactive insulin concentrations, and circulating autoantibodies against endogenous insulin. Since its original description by Yukimasa Hirata and colleagues in 1970, more than 300 cases have been reported, predominantly in the Japanese population (92). The distinction between clinically silent macro-insulinemia and IAS is not always clear. It has been proposed that the clinical phenotype depends largely on the affinity and binding capacity of the insulin autoantibodies. In IAS, low-affinity, high-capacity antibodies allow intermittent dissociation of insulin from the immune complexes, resulting in recurrent episodes of hypoglycemia (93). In contrast, more stable insulin–autoantibody complexes may produce little or no biologically available insulin and manifest primarily as immunoassay interference without clinically significant hypoglycemia.

This distinction has important diagnostic implications. PEG precipitation removes both stable macro-insulin complexes and insulin–autoantibody complexes present in IAS. Therefore, low insulin recovery after PEG precipitation cannot reliably differentiate clinically silent macro-insulinemia from IAS. Although asymptomatic IAS has been reported only exceptionally (16), patients with insulin autoantibodies should be interpreted cautiously, as clinically significant hypoglycemia may develop during follow-up.

In summary, macro-insulin should be considered in patients with markedly elevated insulin concentrations that are discordant with the clinical presentation, glycemic status, and other biochemical findings. Because PEG precipitation cannot distinguish macro-insulin from IAS, laboratory results should always be interpreted in conjunction with the clinical picture and, whenever available, insulin autoantibody testing.

### 2.7 Macro-calcitonin

Macro-calcitonin is a rare macrohormone formed by complexes between calcitonin (CT) and circulating immunoglobulins, resulting in immunoassay interference and falsely elevated serum CT concentrations. This phenomenon is of particular clinical importance because CT is the principal biochemical marker used for the diagnosis and postoperative surveillance of patients with medullary thyroid carcinoma (MTC) (11). Following surgical resection of MTC, persistently elevated or increasing CT concentrations usually indicate residual disease, locoregional recurrence, or distant metastases and often prompt further imaging studies or additional therapeutic interventions. However, in rare cases, elevated CT concentrations may instead reflect the presence of macro-calcitonin.

To date, only three cases of macrocalcitoninemia identified during postoperative follow-up of patients with MTC have been reported (11). In all cases, PEG precipitation demonstrated extremely low CT recovery (2–7%), strongly suggesting the presence of a macrocomplex, while GFC confirmed a high-molecular-weight calcitonin-containing complex.

The differential diagnosis of hypercalcitoninemia should primarily include MTC, chronic kidney disease, and other recognized causes of CT elevation. However, when CT concentrations are discordant with the clinical presentation, imaging findings, or postoperative course, macro-calcitonin should also be considered. Recognition of this rare source of immunoassay interference may prevent unnecessary imaging, invasive diagnostic procedures, inappropriate treatment, and considerable patient anxiety.

## 3 Discussion

Macrohormones represent a heterogeneous but conceptually unified group of high-molecular-weight hormone-immunoglobulin complexes that interfere with immunoassay-based hormone measurements while exhibiting little or no biological activity. As demonstrated across the spectrum of hormones reviewed here, the presence of macrohormones may lead to striking discordance between laboratory results and the patient’s clinical presentation. Recognition of this phenomenon is therefore essential for accurate diagnosis and appropriate clinical decision-making in endocrinology.

A unifying feature of all macrohormones is their capacity to cause persistent hormone elevation in routine immunoassays despite preserved target-organ function. This biochemical profile often mimics clinically significant endocrine disorders, including hyperprolactinemia, subclinical hypothyroidism, primary ovarian insufficiency, hyperparathyroidism, acromegaly, ACTH-dependent Cushing syndrome, or hyperinsulinemic states. Therefore, macrohormone-related assay interference has repeatedly resulted in unnecessary diagnostic procedures, such as advanced imaging, dynamic endocrine testing, genetic analyses, and even surgical interventions, as well as inappropriate pharmacological treatment.

From a mechanistic perspective, most macrohormones described to date consist of single-chain peptide hormones (ACTH, GH, prolactin, insulin, and calcitonin) or glycosylated α/β heterodimeric glycoproteins (TSH, FSH, LH) bound to circulating immunoglobulins, predominantly IgG, although other isotypes have been reported. These complexes are characterized by reduced clearance and prolonged circulatory persistence, explaining their accumulation and detectability in immunoassays. Importantly, the formation of macrohormones does not appear to follow a single pathogenic pathway. Proposed mechanisms include autoantibody production, altered immune tolerance, and post-translational hormone modifications, but definitive triggers remain largely unknown. This mechanistic heterogeneity likely contributes to the variable clinical contexts in which macrohormones are encountered.

The diagnostic challenge posed by macrohormones is compounded by the limitations of currently available laboratory methods. Polyethylene glycol precipitation remains the most widely accessible screening tool and is effective in identifying high-molecular-weight hormone complexes in clinical practice. However, PEG precipitation does not directly confirm the molecular identity of macrohormones and may yield false-positive or false-negative results depending on the hormone analyzed and assay characteristics.

Gel filtration chromatography is considered the reference method for definitive confirmation, yet its limited availability, cost, and technical complexity restrict routine use. As a result, many reported cases rely on indirect evidence of macrohormone interference, highlighting the need for standardized diagnostic algorithms and improved laboratory accessibility.

In some cases, macrohormones may coexist with an excess or deficiency of the biologically active monomeric hormone, further complicating result interpretation. Moreover, spontaneous dissociation of the hormone from immunoglobulins may trigger transient clinical manifestations, as best documented in IAS. Accordingly, some authors have advocated assessing not only the PEG recovery ratio but also the absolute post-PEG hormone concentration. Therefore, laboratory findings must always be integrated with clinical assessment, imaging results, and complementary biochemical markers. Close collaboration between clinicians and laboratory specialists is essential to ensure appropriate interpretation of discordant results and to avoid premature diagnostic closure.

Based on the available evidence, we propose a practical diagnostic algorithm for the evaluation of suspected macrohormones (**Figure 2**). The algorithm begins with recognition of persistent clinicobiochemical discordance, followed by systematic clinical and laboratory evaluation to exclude physiological causes and common analytical interferences. When unexplained discordance persists, PEG precipitation is recommended as a screening test, with interpretation based primarily on the absolute post-PEG hormone concentration rather than percentage recovery. GFC, the reference method for macrohormone detection, may be considered for confirmation. If macrohormone is excluded, patients should undergo standard hormone-specific evaluation for true hormone excess.

Despite increasing recognition, macrohormones remain underdiagnosed, in part due to inconsistent nomenclature, limited awareness outside specialized endocrine centers, and the absence of universally accepted screening recommendations for most hormones. With the exception of macroprolactin, for which professional societies provide guidance, detection of other macrohormones relies largely on clinical suspicion. This review underscores the need for broader education regarding immunoassay interference, particularly as hormone testing continues to expand across multiple medical specialties.

Finally, substantial gaps in knowledge remain. The true prevalence of most macrohormones is unknown, their molecular structure is incompletely characterized, and their long-term clinical significance beyond assay interference has not been systematically studied. Most available data derive from isolated case reports or small case series, limiting generalizability. Large-scale studies are needed to define epidemiology, elucidate mechanisms of formation, and determine whether selected macrohormones may exert subtle biological effects under specific conditions.

In conclusion, macrohormones constitute an important and underrecognized source of diagnostic error in endocrinology. Awareness of this phenomenon, coupled with a structured approach to discordant hormone results, can prevent misdiagnosis, avoid unnecessary interventions, and improve patient safety. Future research should focus on standardizing diagnostic strategies, expanding access to confirmatory laboratory techniques, and clarifying the clinical implications of these intriguing macromolecular complexes.
